# New β-Carotene-Chitooligosaccharides Complexes for Food Fortification: Stability Study

**DOI:** 10.3390/foods9060765

**Published:** 2020-06-10

**Authors:** Alma Bockuviene, Jolanta Sereikaite

**Affiliations:** 1 Department of Chemistry and Bioengineering, Vilnius Gediminas Technical University, 10221 Vilnius, Lithuania; alma.bockuviene@chf.vu.lt; 2 Department of Polymer Chemistry, Institute of Chemistry, Vilnius University, 01513 Vilnius, Lithuania

**Keywords:** β-carotene-chitooligosaccharides complexes, thermal and long-term stability, UV irradiation, degradation kinetics, colour changes

## Abstract

The application of β-carotene in food industry is limited due to its chemical instability. The drawback may be overcome by designing new delivery systems. The stability of β-carotene complexed with chitooligosaccharides by kneading, freeze-drying and sonication methods was investigated under various conditions. The first-order kinetics parameters of the reaction of β-carotene degradation were calculated. The complexation improved the stability of β-carotene at high temperatures and ensured its long-term stability in the dark at 4 °C and 24 °C, and in the light at 24 °C. In water solutions, the best characteristics were exhibited by the complexes prepared by freeze-drying and sonication methods. In the powder form, the complexes retained their colour for the period of the investigation of four months. The calculated total colour differences of the complexes were qualified as appreciable, detectable by ordinary people, but not large. Therefore, β-carotene-chitooligosaccharides complexes could be used as a new delivery system suitable for food fortification.

## 1. Introduction

β-Carotene is a natural pigment belonging to the carotenoids family. It is composed of a polyene system with eleven conjugated double bonds and a β-ring at each end of the chain. Humans are unable to synthesize β-carotene in their bodies. Thus, it is obtained from the diet. β-Carotene is a precursor of vitamin A and has other biological activities that contribute to human health [1,2]. It exhibits high antioxidant activity [3]. The consumption of β-carotene is also associated with the reduced risk of cardiovascular diseases [4,5], cancer [6,7], type 2 diabetes [8,9] and the prevention of age-related macular degeneration [10,11]. Therefore, there is an increasing interest in the fortification of food with β-carotene. However, β-carotene is insoluble in water and has low chemical stability [12,13]. Therefore, its application is limited especially in water-based food.

The encapsulation of β-carotene using various techniques and materials is a first choice to overcome these drawbacks [14,15,16]. Emulsion-based systems, i.e., nano/microemulsions of oil-in-water type are one of the most investigated and utilized colloidal systems for β-carotene encapsulation and delivery [17,18]. Recently, an effective encapsulation technology based on amylose inclusion complex with amphiphilic compound has been proposed and used for the incorporation of β-carotene as a guest molecule. The amylose-surfactant-β-carotene and amylose-ascorbyl palmitate-β-carotene ternary systems have been developed, and the stability of encapsulated β-carotene has been improved [19]. The encapsulation of β-carotene within apoferritin nanocages can also be regarded as inclusion complexation. The thermal stability of encapsulated β-carotene has been markedly improved. Moreover, such carotenoid-containing nanocomposites are water-soluble [20].

Previously, we prepared β-carotene-chitooligosaccharides complexes (CAR-CHIOS) by the mechanochemical methods, i.e., kneading, freeze drying and sonication [21]. To the best of our knowledge, the synthesis of CAR-CHIOS complexes was presented for the first time. The highest complexation yield and water solubility and the lowest hydrodynamic radius were found for complexes prepared by freeze-drying and sonication methods. Moreover, we determined that the complexation did not cause the loss of the antioxidant activity of β-carotene. The carotenoid was complexed with a carrier which itself has biological activities and is considered as a component of functional food [22]. The application of CAR-CHIOS complexes can result in the synergistic effect. CHIOS exhibit anti-oxidant, anti-inflammatory and anti-cancer activities [22,23], positively affect probiotic bacteria [24] and have antimicrobial activity [25]. Moreover, chitosan, from which CHIOS are produced by enzymatic or chemical hydrolysis is approved as a food additive and introduced in the European Pharmacopeia and the US National Formulary in 2008 and 2011, respectively. Taken together, CAR-CHIOS complexes could be in future a new technology for β-carotene delivery. [26]. However, for the application of CAR-CHIOS complexes, the knowledge on its stability under various conditions is of the primary importance. The complexation of carotenoids with carbohydrates by mechanochemical methods are not largely investigated and the impact of this techniques on the properties of carotenoids are not fully understood. Previously, Polyakov et al. [27] complexed mechanochemically canthaxanthin with arabinogalactan and the water solubility of the carotenoid was increased.

Therefore, this study aims for the evaluation of the effect of temperature, pH and UV irradiation on CAR-CHIOS complexes prepared by mechanochemical methods. The changes of the colour of complexes in the form of powder under the long-term storage conditions were also presented.

## 2. Materials and Methods

### 2.1. Materials

β-carotene was purchased from Sigma-Aldrich. Chitooligosaccharides (CHIOS) were prepared by the acid hydrolysis of chitosan (M_w_ 500,000 Da) as previously described [21] The average molecular weight of obtained chitooligosaccharides was 3.8 kDa, and the degree of deacetylation was 80%. All other reagents were of analytical grade.

### 2.2. Synthesis of β-Carotene-Chitooligosaccharides Complexes

CAR-CHIOS complexes were prepared as previously described by Bockuviene and Sereikaite [21]. Briefly, the complexes named as LF4 and LF5 were obtained by the freeze-drying method. For LF4 synthesis, the suspension prepared from10 mg of CAR in 0.5 mL of ethanol and 10 mg of CHIOS in 1 mL water were mixed using a magnetic stirrer for 48 h at 25 °C under dark conditions. For LF5 preparation, instead of water, CHIOS were dissolved in 1 mL of acidic water solution (pH 4). Finally, the solvents were evaporated in an oven at 30 °C, and obtained fine powder was dissolved in 5 mL of deionised water, frozen at −20 °C and lyophilised.

The complexes named as S6 and S7 were prepared by the sonication method. For S6 synthesis, 10 mg of CAR in 0.5 mL of ethanol and 10 mg of CHIOS were dissolved in 1 mL of ethanol/water solution (70/30, *v*/*v*) under magnetic stirring for 24 h in the dark at 25 °C. For S7 synthesis, 10 mg of CAR in 0.5 mL of ethanol and 10 mg of CHIOS in 1 mL acidic water solution (pH 4) were mixed. Subsequently, the solutions were sonicated using an ultrasound probe at 100 W for 30 min, pulse treatment 30:30 on/off, and 60% amplitude. Finally, the complexes paste were dried in an oven at 30 °C for 24 h and pulverised into a fine powder.

The complex named as KD3 was prepared by the kneading method. For KD3 synthesis, 10 mg of CAR and 10 mg of CHIOS were mixed in a ceramic mortar. Thereafter, the mixture was dissolved in 1 mL of degassed water/ethanol (70/30, *v*/*v*) to obtain the paste, followed by grinding for 30 min. The resulting paste was dried in an oven at 30 °C for 24 h and pulverised into a fine powder.

All CAR-CHIOS complexes were dissolved in water and the solutions were centrifuged at 3000 rpm (IKA mini G centrifuge, Staufen Germany) for 15 min to separate unreacted CAR and filtered through 0.45 μm membrane filter. The solutions were dried in an oven at 30 °C for 24 h. All complexes prepared by three different techniques were stored in a refrigerator and protected from light.

### 2.3. Stability of β-Carotene Complexed with CHIOS

For the investigation of thermal and pH stability of β-carotene, CAR-CHIOS complexes (1 mg/mL) were dissolved in water and the pH was adjusted to the desired final value (3, 5, 7 or 8) using either NaOH or HCl solution. For thermal stability experiments, the samples of 1 mL were transferred into glass tubes with caps and incubated in the dark at 40 °C, 60 °C, 80 and 100 °C for 30 min. Then, the samples were cooled down in the ice bath to stop the reaction. For the investigation of pH stability, the samples of 1 mL into glass tubes with caps were stored in the dark at 24 °C and 4 °C and in the light at 24 °C for 30 days. For the investigation of the effect of UV irradiation on the chemical degradation of β-carotene in the complexes, the solution of CAR-CHIOS complexes (1 mg/mL) was prepared at different pH values (3, 5, 7 and 8) and exposed to the irradiation produced by a UVC lamp (Philips, Amsterdam, Holland, 254 nm, 15 W) at the distance of 35 cm in the dark at 24 °C. The samples were analysed after 15, 30, 120 and 240 min of UV irradiation.

In all stability experiments, the chemical degradation of β-carotene was monitored by the registration of absorbance at 450 nm [28]. All stability experiments were performed in triplicate, and the relative stability was calculated using the following equation: Relative stability (%) = (A_t_)/A_0_) × 100, where A_0_ and A_t_ are the absorbance of CAR-CHIOS solution at the initial moment and at the time t, respectively. For the calculation of the first-order rate constant of the degradation reaction (k) and reaction half-life (*t*_1/2_), the data were fitted to the Equation (1) using the SigmaPlot 14 software, where c_t_ is β-carotene concentration at the time t under the storage conditions (in the dark at 4 °C and 24 °C, in the light at 24 °C or under UV irradiation at 254 nm) and c_o_ is the initial concentration of β-carotene.

c_t_ = c_o_exp(−kt) (1)

Dynamic light scattering method (DLS) was used for the evaluation of changes of hydrodynamic diameter of CAR-CHIOS complexes under different storage conditions. The hydrodynamic diameter of CAR-CHIOS complexes was measured using *Zetasizer Nano ZS* (Malvern Instruments) equipped with a 4 mW HeNe laser at a wavelength of 633 nm. The measurements of the intensity of scattered light were performed at 25 °C and at an angle of 173°. The size distribution data were analysed by the Malvern Zetasizer software 7.03 (Malvern Panalytical, Malvern, UK).

### 2.4. Colour Measurement

The chromatic characteristics of samples under the storage in the dark at 4 °C were evaluated using a hand-held spectrophotometer Konica Minolta CM-700d. The CIELAB colour space coordinates L* (for the lightness from black (0) to white (100)), a* (from green (−) to red (+)), b* (from blue (−) to yellow (+)) were measured using the illuminant D_65_. The spectrophotometer was calibrated against a white background. Then, the samples were placed onto a transparent glass plate with white background, and colour measurements were performed in triplicate. The colour assessment was performed periodically during 120 days. The Chroma or colour saturation (C*_ab_), Hue angle (h*_ab_) and the total colour difference (ΔE*_ab_) were calculated as previously described [29].

### 2.5. Statistical Analysis

Data are presented as mean ± standard deviation (*n* = 3). One-way analysis of variance (ANOVA, *p* < 0.05) was used to compare the data and define statistically significant result.

## 3. Results and Discussion

### 3.1. The Effect of Temperature on β-Carotene-Chitooligosaccharides Complexes

**Figure 1:**
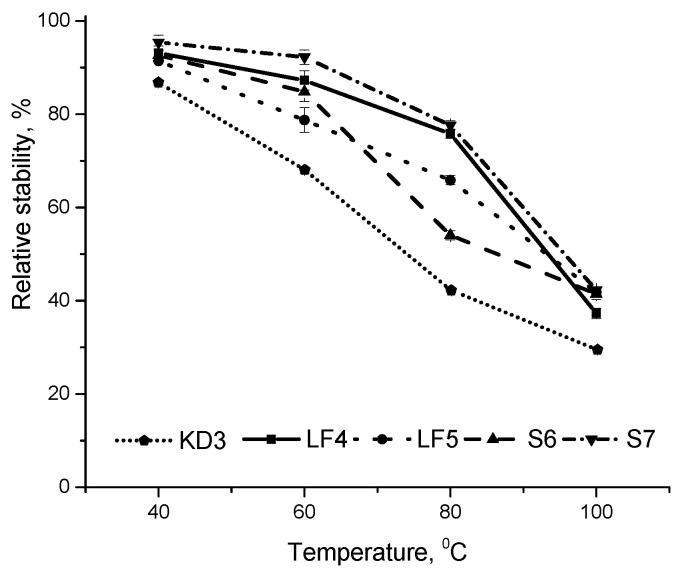
Thermal stability of CAR during thermal treatment of CAR-CHIOS complexes KD3, LF4, LF5, S6 and S7 in the dark for 30 min at various temperatures and neutral pH. Each value was expressed as mean ± standard deviation (*n* = 3).

About 60% of CAR complexed with CHIOS degraded under the storage at 100 °C for 30 min (Figure 1). Moreover, the increase in particle hydrodynamic diameter about of 20% comparing with fresh prepared complexes was registered. The most likely reason of that is the aggregation of degraded complexes and the changes in Van der Waals interactions in CHIOS molecules [22]. At 40 °C and 60 °C the complexes were more stable and the loss of CAR was about 20%. Moreover, the temperature stability of β-carotene complexed with CHIOS was not practically dependent on pH value of solution. Previously, the thermal degradation of 0.1% (*w*/*v*) β-carotene dissolved in ethyl acetate was investigated [28]. After the incubation of solution at 60 and 80 °C for 30 min, about 50 and 20% of β-carotene retained, respectively. Therefore, the complexation of β-carotene with CHIOS obviously increases its thermal stability (Figure 1). The degradation of β-carotene at high temperatures is mainly due to oxidation [30]. Supposedly, for the complex formation, CAR entraps into CHIOS chains being helical in shape, and the supramolecular system of the complexes forms [21]. The three-dimensional network of matrix can act as a physical barrier protecting β-carotene. Moreover, CHIOS have an antioxidant activity [31] and can prevent β-carotene from oxidation. However, KD3 complex prepared by the kneading method exhibited significantly lower stability at the higher temperature comparing with the complexes LF4, LF5 and S6, S7 prepared by freeze-drying and sonication methods, respectively.

### 3.2. The Effect of pH and Temperature on the Long-Term Stability of Complexed β-Carotene

**Figure 2:**
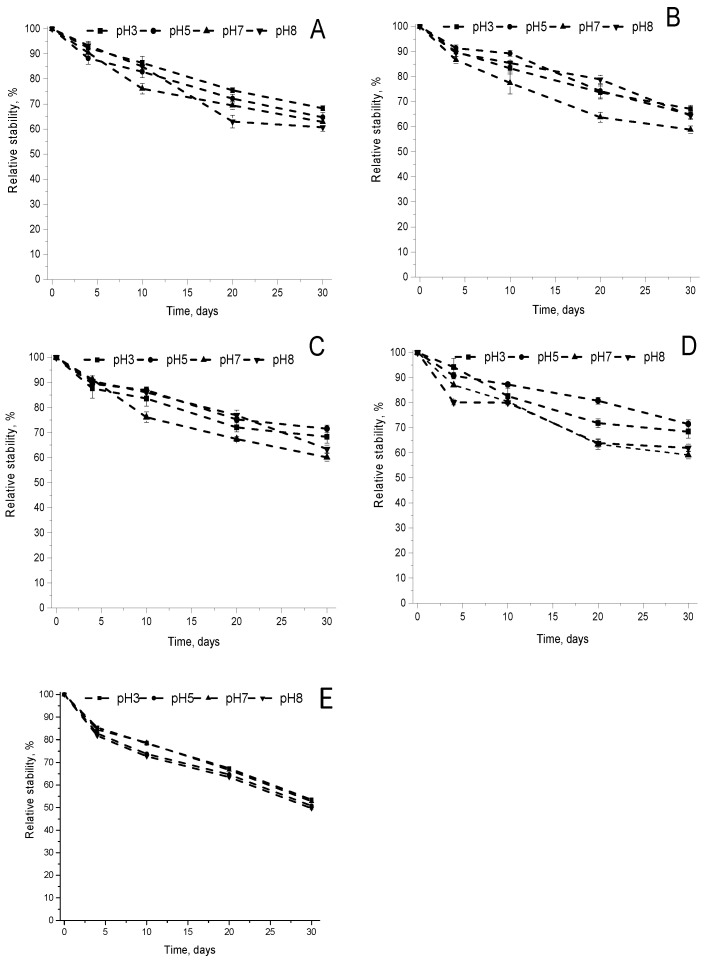
The effect of pH on CAR stability during the storage of CAR-CHIOS complexes LF4 (**A**), LF5 (**B**), S6 (**C**), S7 (**D**) and KD3 (**E**) in the dark at 4 °C. Each value was expressed as mean ± standard deviation (*n* = 3).

The pH influence on the long-term stability of CAR complexed with CHIOS was investigated under the storage in the dark at 4 °C and 24 °C and in the light at 24 °C (Figure 2, Figures S1 and S2). The first-order rate constants of the reaction of β-carotene degradation and the half-lives were calculated under different conditions of the storage (Table 1). The applicability of the first-order kinetics model for β-carotene degradation was previously reported [32,33]. Under the storage in the dark at 4 °C, the kinetics parameters of β-carotene degradation were significantly dependent on the pH values. The lowest stability was determined for KD3 complex at all pH values comparing with the complexes prepared by freeze-drying and sonication methods. The complexes S6 and S7 exhibited the highest stability at pH 5. Obtained results are very important having in mind the possible application of complexes as CAR delivery system for the fortification of food with various pH values. The sensitivity of carotenoids to acids is related with the formation of carbocations of carotenoid molecules [34]. Chitooligosaccharides molecules having the protonated amino groups at the acid and slightly acid environment [35] probably create an electrostatic barrier for the protonation of a carotenoid molecule. The size of complexes and polydispersity index under all storage conditions for 30 days also changed insignificantly. In the dark, all complexes were less stable at 24 °C than at 4 °C temperature of storage. At 24 °C, the changes of the stability of complexes LF4 and LF5 were insignificant in the pH range 3–8. The complexes S6 and S7 exhibited the highest stability at pH 3, and the differences in the stability were insignificant in the pH range 5–8. It seems that in the dark at 24 °C the degradation of β-carotene is more influenced by the temperature of storage than by pH value.

Under the storage in the light at 24 °C (Figure S2), the KD3 complex prepared by the kneading method exhibited the lowest stability, which differed insignificantly in the pH range 3–8. Overall, the half-lives of all complexes decreased comparing with the storage conditions in the dark at 24 °C. The complexes LF4 and LF5 prepared by freeze-drying method were unexpectedly more stable at the acid or slightly acid environment than at the neutral or slightly alkali medium. Overall, in the light at 24 °C the half-lives of β-carotene degradation in all our complexes were in the range of 9.83–26.87 days, while the half-life of β-carotene dissolved in ethyl acetate was 18.16 h at 21 °C [28]. The stabilization of β-carotene by the complexation with CHIOS is obvious.

### 3.3. The Effect of UV Irradiation on β-Carotene-Chitooligosaccharides Complexes

The most significant effect of UV irradiation on β-carotene degradation was found for the KD3 complex (Table 1, Figure S3). The complexes LF4 and LF5 obtained by the freeze-drying method were most stable, and the half-lives of β-carotene degradation were the longest. The degradation rate of β-carotene complexed with CHIOS was similar at all pH values for all complexes with the exception for the complexes S6 and S7 obtained by the sonication method. At the acid conditions (pH 3), β-carotene in that complexes degraded significantly faster comparing with other pH values. As can be seen, the complexes obtained by freeze-drying techniques were significantly more stable than by the sonication method. UV irradiation causes the production of free radicals and induces photochemical oxidation of compounds. CHIOS that wrap CAR are known as compounds having an antioxidant activity. Those antioxidant properties are closely related to the molecular mass of CHIOS and their amino and hydroxyl groups [22,31,36]. It is plausible that the sonication method induces changes in CHIOS molecules, and in consequence, the protective effect against the damage of CAR caused by UV irradiation reduces. The lower stability of β-carotene in KD3 complex under UV irradiation conditions as well under the long-term storage in the dark and in the light is probably also related to the changes in CHIOS structure induced by kneading. Therefore, freeze-drying method for complex preparation ensures the highest stability of β-carotene against the degradation by UV irradiation.

### 3.4. The Changes of Colour Parameters of CAR-CHIOS Complexes

**Figure 3:**
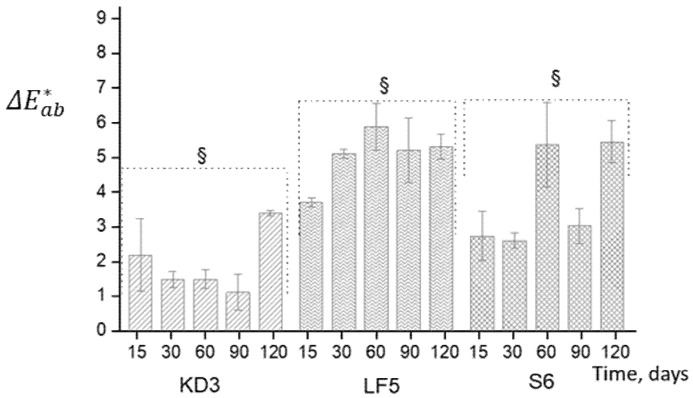
The total colour differences of CAR-CHIOS complexes KD3, LF5, S6 during the storage for 120 days in the dark at 4 °C. Each value was expressed as mean ± standard deviation (*n* = 3). Significant (§) differences of the values of colour parameter are determined using one-way analysis of variance (ANOVA, *p* < 0.05).

The changes of colour parameters of complexes during their storage in the dark at 4 °C were monitored by non-destructive method measuring colour fading. The data are presented in Table 2 and Figure 3.

As can be seen, the colour parameters of CAR-CHIOS complexes are different and depend on the methods of complexes preparation. As follows from the ratio a*/b* and Hue angle, the colours of LF5 and S6 complexes are shifted to orange-red-orange tones, and the colour of KD3 is more orange-yellow [37]. During four months of storage the changes of colour parameters of the complexes are significant. However, the total colour differences ΔE*_ab_ are in the interval of 3.0–6.0 and qualified as appreciable, detectable by ordinary people, but not large [38]. It shows the utility of the complexation in the protection of β-carotene and the retention its colour.

## 4. Conclusions

To sum up, the complexation of β-carotene with chitooligosaccharides improved the temperature stability and ensured its long-term stability. In water solutions, the best characteristics were exhibited by the complexes prepared by freeze-drying and sonication methods. In the powder form, the complexes retained their colour for the period of the investigation of four months. The calculated total colour differences of the complexes were qualified as appreciable, detectable by ordinary people, but not large. Therefore, β-carotene-chitooligosaccharides complexes, which both components are approved as food additives, could be in future a new technology for β-carotene delivery and applied as a new formulation in food systems.

## Figures and Tables

**Table 1:** First-order kinetics parameters for CAR-CHIOS complexes degradation under different conditions ^1,2,3^.

Storage Conditions	Sample	pH							
		3	5	7	8	3	5	7	8
		*k* × 10^3^, day^−1^				*t*_1/2_, days			
Dark at	KD3	14.38 ± 0.26 ^aA^	14.56 ± 0.29 ^aB^	16.47 ± 0.48 ^aC^	17.61 ± 0.37 ^aD^	48.83 ± 0.57	47.25 ± 0.69	42.76 ± 0.35	39.45 ± 0.16
4 °C	LF4	6.34 ± 0.08 ^bA^	8.24 ± 0.06 ^bB^	5.29 ± 0.07 ^bC^	5.83 ± 0.09 ^bD^	109.34 ± 0.54	85.64 ± 0.39	133.88 ± 0.56	118.36 ± 0.28
	LF5	6.76 ± 0.04 ^cA^	8.33 ± 0.05 ^bB^	5.09 ± 0.07 ^bC^	5.39 ± 0.03 ^cD^	102.46 ± 0.24	85.34 ± 0.65	120.33 ± 0.29	128.43 ± 0.73
	S6	6.15 ± 0.05 ^dA^	2.46 ± 0.04 ^cB^	4.88 ± 0.01 ^cC^	5.18 ± 0.32 ^dD^	122.45 ± 0.93	125.34 ± 0.46	140.43 ± 0.73	132.65 ± 0.75
	S7	6.23 ± 0.02 ^dA^	2.47 ± 0.05 ^cB^	4.88 ± 0.08 ^cC^	5.18 ± 0.08 ^dD^	118.75 ± 0.31	129.46 ± 0.65	145.56 ± 0.22	138.45 ± 0.82
		*k* × 10^2^, day^−1^				*t*_1/2_, days			
Dark at	KD3	2.84 ± 0.07 ^aA^	3.91 ± 0.08 ^aB^	2.95 ± 0.04 ^aA^	3.91 ± 0.03 ^aB^	24.59 ± 0.48	17.74 ± 0.19	23.94 ± 0.59	17.38 ± 0.24
24 °C	LF4	2.39 ± 0.05 ^bA^	2.36 ± 0.12 ^bA^	2.45 ± 0.04 ^bA^	2.33 ± 0.05 ^bA^	27.67 ± 0.36	27.06 ± 0.43	28.24 ± 0.59	29.84 ± 0.63
	LF5	2.49 ± 0.05 ^bA^	2.49 ± 0.06 ^bA^	2.35 ± 0.04 ^bA^	2.23 ± 0.06 ^bA^	27.56 ± 0.79	28.16 ± 0.73	29.18 ± 0.28	30.52 ± 0.22
	S6	1.89 ± 0.01 ^cA^	2.59 ± 0.06 ^bB^	2.55 ± 0.04 ^bB^	2.73 ± 0.05 ^cB^	36.04 ± 0.91	26.77 ± 0.74	26.51 ± 0.59	25.48 ± 0.79
	S7	1.76 ± 0.05 ^cA^	2.39 ± 0.04 ^bB^	2.45 ± 0.04 ^bB^	2.43 ± 0.05 ^bB^	35.76 ± 0.70	28.47 ± 0.43	28.75 ± 0.69	28.68 ± 0.76
Light at	KD3	6.97 ± 0.99 ^aA^	6.96 ± 0.58 ^aA^	6.95 ± 0.63 ^aA^	6.97 ± 0.53 ^aA^	9.83 ± 0.48	9.95 ± 0.55	9.98 ± 0.64	9.95 ± 0.46
24 °C	LF4	2.63 ± 0.92 ^bA^	3.05 ± 0.85 ^bB^	4.64 ± 0.87 ^bC^	3.69 ± 0.65 ^bB^	26.73 ± 0.59	22.49 ± 0.68	16.04 ± 0.54	18.78 ± 0.68
	LF5	2.58 ± 0.97 ^bA^	3.08 ± 0.65 ^bB^	4.91 ± 0.24 ^cC^	3.98 ± 0.51 ^bD^	26.87 ± 0.56	22.48 ± 0.68	14.05 ± 0.56	17.31 ± 0.79
	S6	3.06 ± 0.57 ^cA^	3.09 ± 0.84 ^bA^	3.09 ± 0.28 ^dA^	3.05 ± 0.75 ^cA^	22.49 ± 0.77	22.81 ± 0.41	22.78 ± 0.41	22.54 ± 0.43
	S7	3.96 ± 0.68 ^dA^	3.13 ± 0.59 ^bB^	3.15 ± 0.39 ^dB^	4.71 ± 0.59 ^dC^	17.65 ± 0.85	16.76 ± 0.75	16.71 ± 0.86	15.65 ± 0.54
		*k* × 10^3^, min^−1^				*t*_1/2_, min^−1^			
UVC, dark	KD3	25.95 ± 5.84 ^aA^	26.39 ± 2.45 ^aB^	26.76 ± 3.85 ^aC^	25.89 ± 4.85 ^aD^	26.71 ± 0.54	26.45 ± 0.61	25.90 ± 0.46	26.86 ± 0.73
at 24 °C	LF4	6.59 ± 0.49 ^bA^	6.04 ± 0.54 ^bB^	6.68 ± 0.58 ^bA^	6.50 ± 0.64 ^bA^	105.71 ± 0.34	114.45 ± 0.61	103.90 ± 0.46	105.86 ± 0.63
	LF5	12.92 ± 0.45 ^cA^	8.53 ± 0.35 ^cB^	6.64 ± 0.38 ^bC^	6.08 ± 0.66 ^cD^	53.63 ± 0.67	80.98 ± 0.54	105.05 ± 0.59	115.95 ± 0.62
	S6	13.65 ± 0.74 ^dA^	9.19 ± 0.47 ^dB^	9.11 ± 0.46 ^cB^	9.09 ± 0.66 ^dB^	50.76 ± 0.73	75.96 ± 0.65	75.28 ± 0.86	76.42 ± 0.87
	S7	15.82 ± 0.53 ^eA^	9.16 ± 0.79 ^dB^	9.18 ± 0.77 ^cB^	9.18 ± 0.75 ^dB^	43.80 ± 0.51	75.61 ± 0.68	75.58 ± 0.42	75.31 ± 0.54

^1^ Data are presented as mean ± standard deviation (*n* = 3). ^2^ The data were fitted to first-order kinetics, the coefficient of correlation *R* was in the interval of 0.8286–0.9994. ^3^ Different capital letters represent significant differences in the mean within the row and different lowercase letters represent significant differences within the column (*p* < 0.05).

**Table 2:** The colour parameters of CAR-CHIOS complexes during the storage of 120 days in the dark at 4 °C ^1^.

Sample	Time, Days	L*	a*	b*	a*/b*	C*_ab_	*h* *_ab_
		**§**	**§**	**§**	**§**	**§**	**§**
KD3	0	65.44 ± 0.48	4.24 ± 0.3	24.59 ± 0.42	0.17 ± 0.01	24.96± 0.46	80.23 ± 0.56
	15	63.75 ± 0.26	3.86 ± 0.18	25.41 ± 0.33	0.15 ± 0.01	25.70 ± 0.49	81.31 ± 0.33
	30	64.53 ± 0.39	4.81 ± 0.24	25.56 ± 0.42	0.18 ± 0.01	26.02 ± 0.42	79.34 ± 0.54
	60	64.38 ± 0.45	5.09 ± 0.71	24.26 ± 0.63	0.20 ± 0.02	24.80 ± 0.77	78.12 ± 0.48
	90	64.44 ± 0.02	4.31 ± 0.16	24.52 ± 0.03	0.17 ± 0.01	24.90 ± 0.02	80.02 ± 0.30
	120	63.31 ± 0.01	3.16 ± 0.01	22.22 ± 0.01	0.14 ± 0.02	22.44 ± 0.01	81.83 ± 0.02
		**§**	**§**	**§**	**§**	**§§**	**§**
LF5	0	54.60 ± 0.42	13.49 ± 0.49	14.72 ± 0.14	0.91 ± 0.03	19.97± 0.35	47.50 ± 1.06
	15	57.35 ± 0.25	15.37 ± 0.29	13.25 ± 0.63	1.16 ± 0.04	20.29 ± 0.58	40.76 ± 1.07
	30	58.60 ± 0.33	16.63 ± 0.35	14.46 ± 0.44	1.15 ± 0.02	22.04 ± 0.50	41.01 ± 0.71
	60	59.48 ± 0.95	16.62 ± 0.26	14.79 ± 0.06	1.12 ± 0.02	22.25 ± 0.16	41.68 ± 0.57
	90	58.64 ± 0.32	16.54 ± 0.37	14.48 ± 0.43	1.14 ± 0.03	21.98 ± 0.52	41.37 ± 1.05
	120	57.99 ± 0.77	16.38 ± 0.32	11.89 ± 0.01	1.37 ± 0.02	20.24 ± 0.26	40.67 ± 0.33
		**§**	**§§**	**§**	**§**	**§**	**§**
S6	0	33.46± 0.33	23.73 ± 0.22	16.30 ± 0.25	1.45 ± 0.03	28.79± 0.08	34.48 ± 0.65
	15	35.81 ± 0.13	24.40 ± 0.64	17.26 ± 0.3	1.41 ± 0.09	29.90 ± 0.96	35.31 ± 1.85
	30	34.27 ± 0.50	24.92 ± 0.88	18.39 ± 0.40	1.35 ± 0.07	30.98 ± 0.48	36.43 ± 1.55
	60	37.56 ± 0.37	24.63 ± 0.50	19.58 ± 0.24	1.25 ± 0.04	31.47 ± 0.24	38.48 ± 0.93
	90	34.65 ± 0.66	24.54 ± 0.56	18.82 ± 0.19	1.30 ± 0.04	30.93 ± 0.32	37.50 ± 0.92
	120	38.38 ± 0.92	25.42 ± 0.01	19.98 ± 1.07	1.27 ± 0.07	32.34 ± 0.66	38.15 ± 1.50

^1^ Insignificant (§§) and significant (§) differences of the values of colour parameters in the column are determined using one-way analysis of variance (ANOVA, *p* < 0.05).

## Supplementary Materials

The following are available online at https://www.mdpi.com/2304-8158/9/6/765/s1, Figure S1: The effect of pH on CAR stability during the storage of CAR-CHIOS complexes LF4 (A), LF5 (B), S6 (C), S7 (D) and KD3 (E) in the dark at 24 °C. Each value is expressed as mean ± standard deviation (*n* = 3), Figure S2: The effect of pH on CAR stability during the storage of CAR-CHIOS complexes LF4 (A), LF5 (B), S6 (C), S7 (D) and KD3 (E) in the light at 24 °C. Each value is expressed as mean ± standard deviation (*n* = 3), Figure S3: The stability of CAR during UV irradiation treatment of CAR-CHIOS complexes LF4 (A), LF5 (B), S6 (C), S7 (D) and KD3 (E) at different pH values. Each value is expressed as mean ± standard deviation (*n* = 3).
