# Supplementary material for: New β-Carotene-Chitooligosaccharides Complexes for Food Fortification: Stability Study
Source: Foods. 2020 Jun 10;9(6):765. doi: 10.3390/foods9060765 (PMC7353500; doi:10.3390/foods9060765)
Supplement: Supplementary file 1 [file foods-09-00765-s001.pdf]

Supplementary data

New β-carotene-chitooligosaccharides complexes for food fortification: stability study

Alma Bockuviene [a,b], Jolanta Sereikaite [a,*]

[a] Department of Chemistry and Bioengineering, Vilnius Gediminas Technical University, Vilnius, Lithuania

[b] Department of Polymer Chemistry, Institute of Chemistry, Vilnius University, Vilnius, Lithuania

*To whom correspondence should be addressed:

Jolanta Sereikaite

Department of Chemistry and Bioengineering

Faculty of Fundamental Sciences, Vilnius Gediminas Technical University

Sauletekio al. 11, LT-10223, Vilnius-40, Lithuania

Telephone: +370 5 2744972; e-mail: jolanta.sereikaite@vgtu.lt

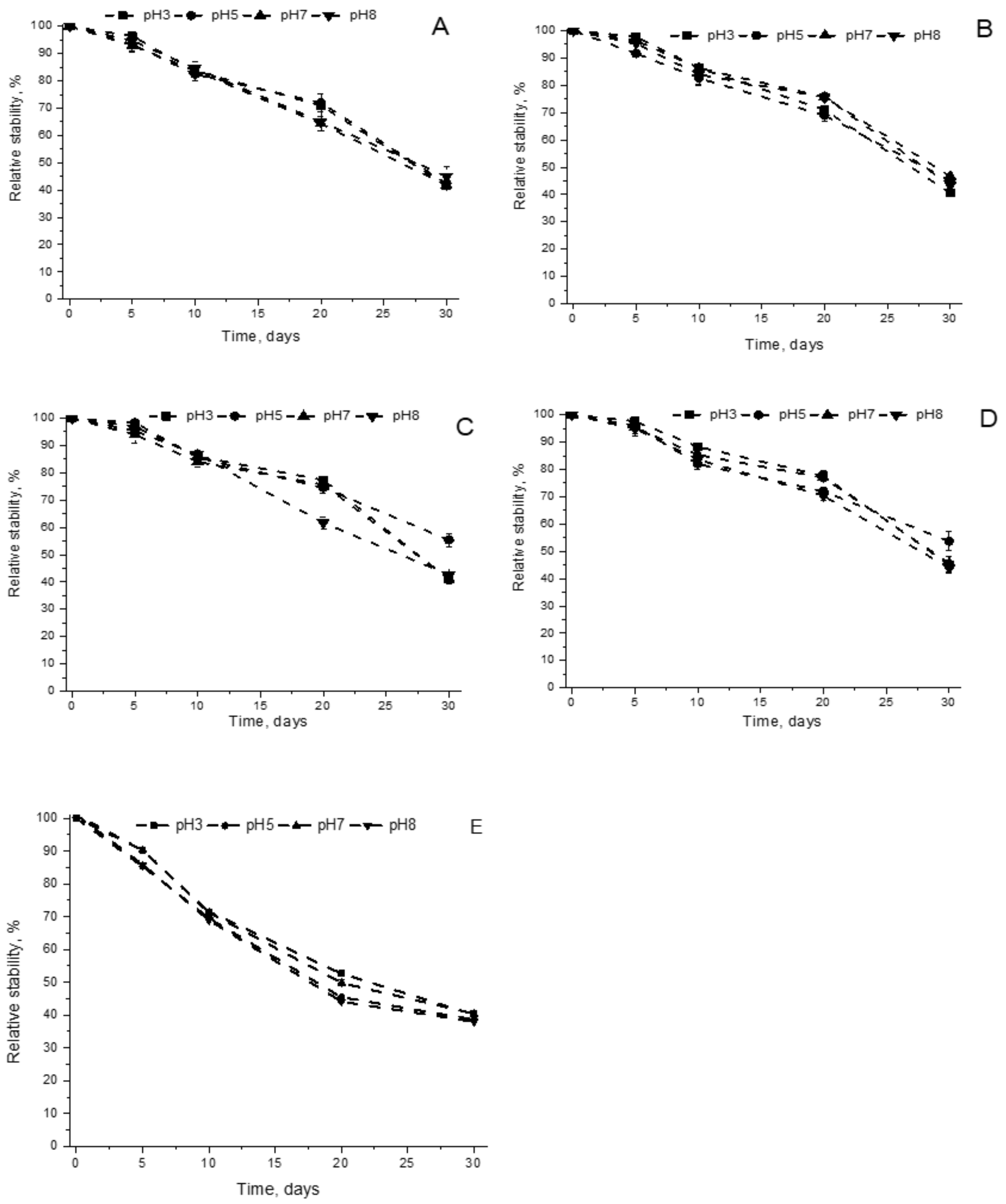

Fig. S1. The effect of pH on CAR stability during the storage of CAR-CHIOS complexes LF4 (A), LF5 (B), S6 (C), S7 (D) and KD3 (E) in the dark at 24 ^{o}C. Each value is expressed as mean ± standard deviation (n=3).

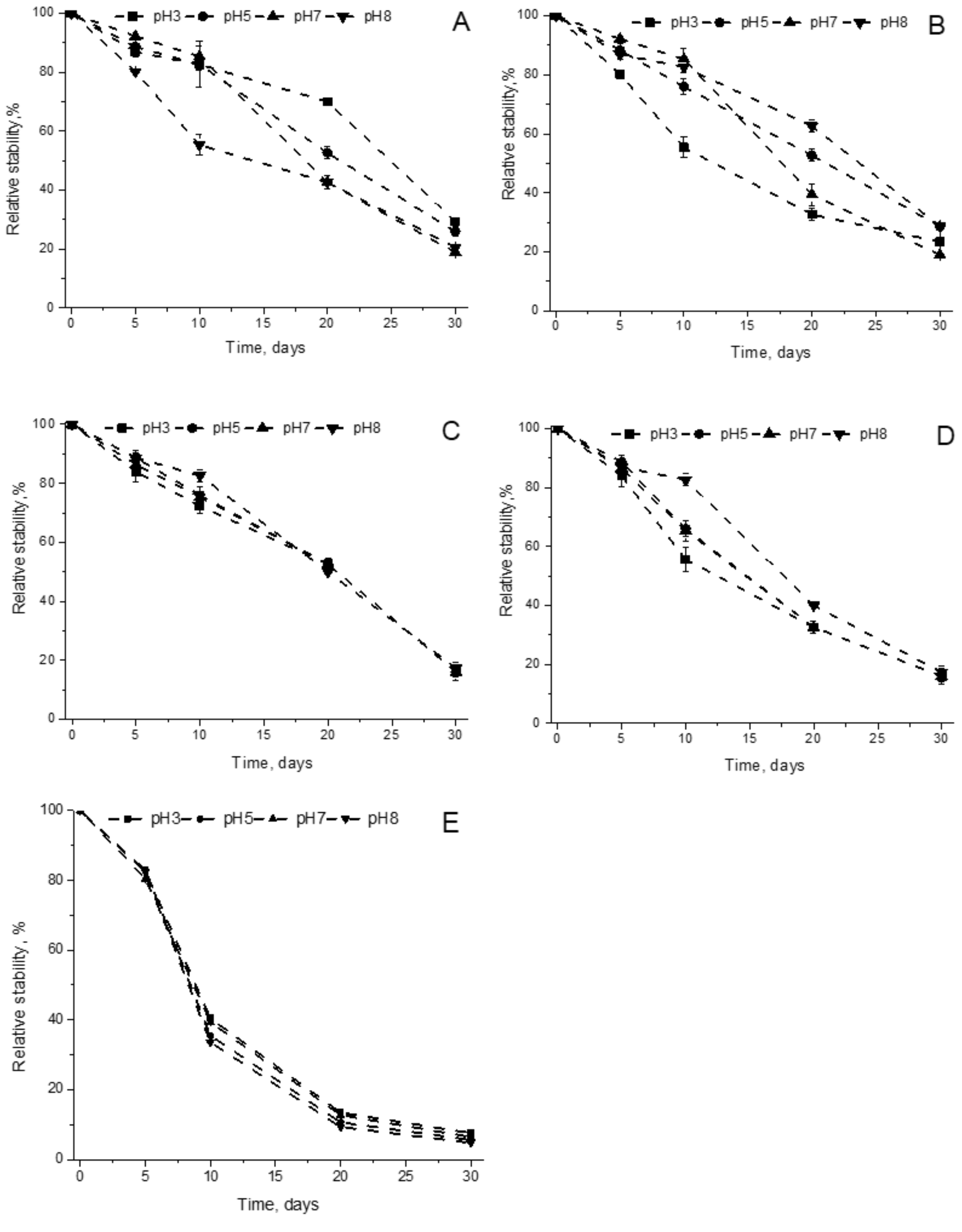

Fig. S2 The effect of pH on CAR stability during the storage of CAR-CHIOS complexes LF4 (A), LF5 (B), S6 (C), S7 (D) and KD3 (E) in the light at 24 °C.· Each value is expressed as mean ±standard deviation (n=3).

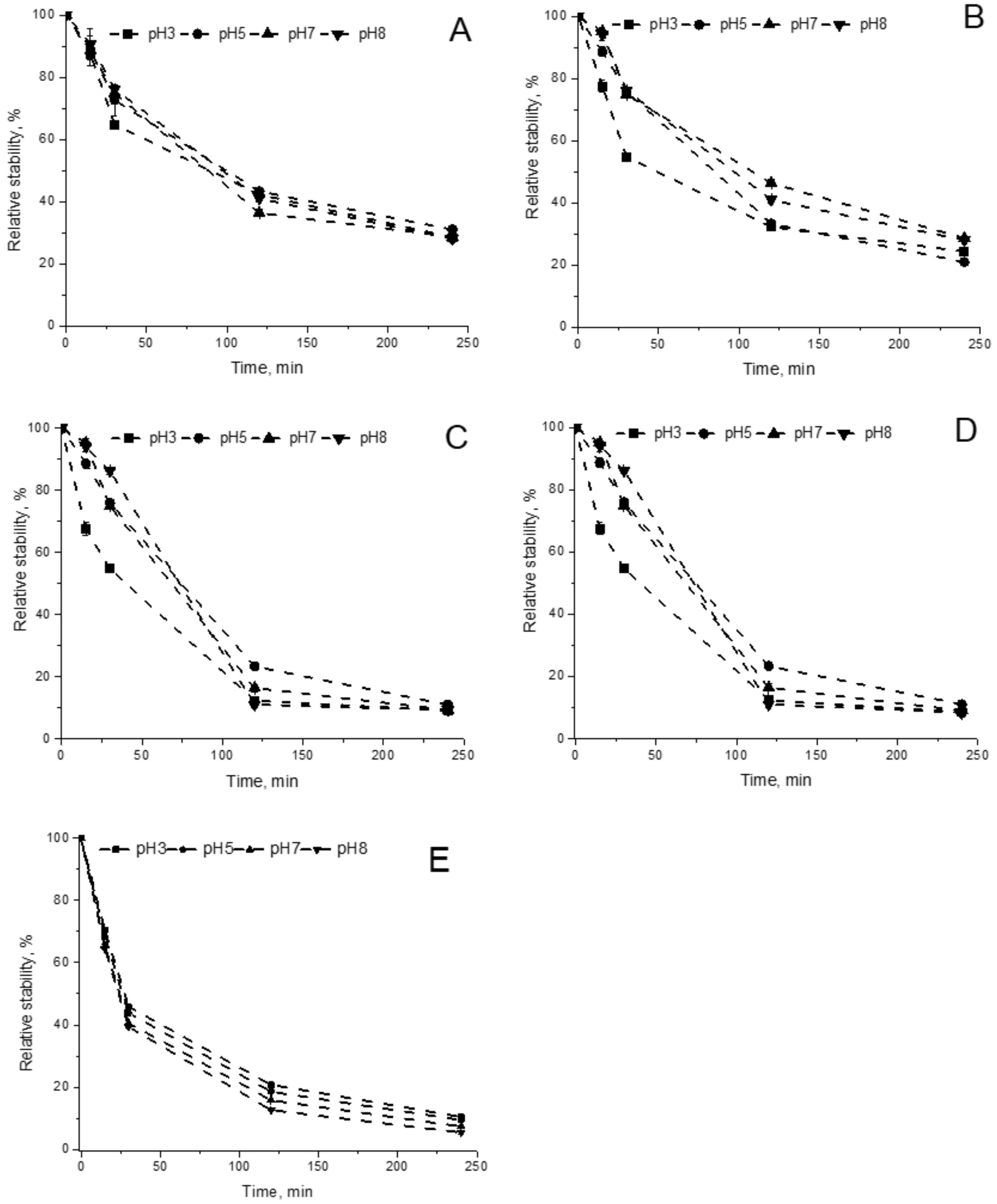

Fig. S3. The stability of CAR during UV irradiation treatment of CAR-CHIOS complexes LF4 (A), LF5 (B), S6 (C), S7 (D) and KD3 (E) at different pH values. Each value is expressed as mean ± standard deviation (n=3).
